# Production, characterization and biomedical potential of biosurfactants produced by haloalkaliphilic archaea from Wadi El-Natrun, Egypt

**DOI:** 10.1186/s12934-024-02351-y

**Published:** 2024-03-14

**Authors:** Basma T. Alghamrawy, Ghada E. Hegazy, Soraya A. Sabry, Hanan Ghozlan

**Affiliations:** 1 Botany & Microbiology Department, Faculty of Science, Alexandria University, Alexandria, Egypt; 2 National Institute of Oceanography & Fisheries, NIOF-Egypt, Alexandria, Egypt

**Keywords:** Halophilic archaea, Biosurfactants, Wound healing, Anticancer, Anti-inflammatory

## Abstract

Extreme halophilic archaea that can live in high saline environments can offer potential applications in different biotechnological fields. This study delves into the fascinating field of halophilic archaea and their ability to produce biosurfactants. Some strains of haloarchaea were isolated from Wadi El-Natrun and were screened for biosurfactants production in a standard basal medium using emulsification index assay. Two strains were chosen as the potential strains for surface tension reduction. They were identified as *Natrialba* sp. BG1 and N3. The biosurfactants production was optimized and the produced emulsifiers were partially purified and identified using FTIR and NMR. Sequential statistical optimization, Plackett–Burman (PB) and Box–Behnken Designs (BBD) were carried out using 5 factors: oil, NaCl, casamino acids, pH, and inoculum size. The most significant factors were used for the next Response Surface Methodology experiment. The final optimal conditions for biosurfactants production were the inoculum size 2% pH 11 and NaCl 250 g/L, for *Natrialba* sp. BG1 and inoculum size 2.2%, pH 10 and NaCl 100 g/L for *Natrialba* sp. N3. The produced biosurfactants were tested for wound healing and the results indicated that *Natrialba* sp. BG1 biosurfactants is more efficient than *Natrialba* sp. N3 biosurfactants. Biosurfactants extracts were tested for their cytotoxic effects on normal cell line as well as on different cancer cells using MTT assay. The findings demonstrated that varying concentrations of the biosurfactants (31.25, 62.5, 125, 250, 500 and 1000 µg/mL) exhibited cytotoxic effects on the cell lines being tested. Additionally, the outcomes unveiled the presence of anti-inflammatory and antioxidant properties for both biosurfactants. Consequently, they could potentially serve as natural, safe, and efficient novel agents for combating cancer, promoting wound healing, and providing anti-inflammatory and antioxidant benefits.

## Introduction

Biosurfactants represent a group of surface-active agents that are naturally produced by various microorganisms, including bacteria and archaea [1, 2]. These exceptional compounds possess both hydrophilic and hydrophobic properties, allowing them to lower the surface tension between different liquid phases. Chemical structures of biosurfactants encompass a wide range, including glycolipids, lipopeptides, lipoproteins, fatty acids, neutral lipids, phospholipids, polymeric and particulate structures. Their commercial potential has been recognized in various industries, such as their use as surfactants for oil recovery and moisturizing agents in cosmetics [3]. Initially discovered during research on hydrocarbon fermentation, biosurfactants were identified as "alternative surfactants" due to their favorable characteristics compared to chemical surfactants. These advantages include low toxicity, biodegradability, effectiveness under extreme conditions of temperature and pH, ecological acceptability, selectivity, and suitability for large-scale production. Moreover, biosurfactants exhibit unique biological activities not found in conventional chemical surfactants [4]. However, a significant challenge lies in the costly large-scale production of biosurfactants, particularly in applications related to petroleum and environmental remediation, recent studies have emphasized the importance of biosurfactants in environmental remediation, enhanced oil recovery, and pharmaceutical formulations. Biosurfactants offer unique properties making them promising candidates in tackling environmental challenges and industrial applications. However, biosurfactant production by halophilic archaea presents challenges. Limited availability of suitable halophilic archaeal strains, extraction and purification difficulties, and the need for genetic manipulation and metabolic engineering hinder large-scale production. Overcoming these obstacles is crucial to optimize biosurfactant production processes and fully exploit the potential of these microorganisms in various applications [5]. Also it is essential to develop processes that utilize waste substrates, thereby reducing environmental impact and achieving cost-effectiveness. The growing need for sustainable and eco-friendly surfactants, coupled with limitations in synthetic surfactant production, has created a significant market demand for biosurfactants. However, conventional production methods have limitations in terms of scalability, cost, and environmental impact. Optimizing biosurfactant production by halophilic archaea can offer a solution to these challenges, providing a more sustainable and efficient approach to meet the industry's needs. Most biosurfactants are complex lipids with high molecular weight, typically produced under highly aerobic conditions, which can be achieved through ex-situ production in aerated bioreactors. Halophilic archaea are microorganisms that thrive in environments with high concentrations of dissolved salt, where sodium chloride (NaCl) serves as a primary requirement for their growth [6]. These microorganisms possess unique physiological and biochemical characteristics, such as their ability to thrive in extreme haloalkaline environments, which make them well-suited for biosurfactant production. Their adaptation to harsh conditions and their potential ecological and industrial relevance, especially in alkaline and saline environments, highlight their significance as promising biosurfactant producers. By incorporating this information, the reader gains a deeper understanding of the novel research direction and the potential impact it can have in addressing specific industry, environmental needs and potential applications in various biotechnological fields [7–10]. This research aims to investigate the production capabilities of biosurfactants by halophilic archaea isolated from extreme habitats in El-Hamra Lake, Wadi El-Natrun, Egypt. The study introduces a novel aspect by exploring the biosurfactants potential of specific microorganisms. To ensure reliability and accuracy, we employ a systematic and rigorous approach in our research. We utilize Plackett–Burman and Box–Behnken experimental designs to thoroughly investigate the production process. This enables us to gather comprehensive data and draw meaningful conclusions.

In addition to studying the production process, our research also focuses on assessing the various activities of the extracted biosurfactants. We specifically evaluate their anti-cancer, antioxidant, anti-inflammatory, and wound healing properties. By examining these diverse aspects, we expand the scope of biosurfactants beyond their traditional applications. Overall, our multifaceted approach allows us to delve deeper into the potential of biosurfactants and explore their broader range of uses.

## Materials and methods

### Source of isolation and screening for biosurfactants producers

Water samples were obtained from El-Hamra Lake, Wadi El-Natrun located on the Cairo-Alexandria Desert Road in Egypt, at coordinates 30.39667°N and 30.31639°E, citing its unique ecological characteristics and potential for housing halophilic archaea. El-Hamra Lake in Wadi El-Natrun is characterized by specific parameters and conditions that make it an intriguing sampling site. The lake’s high salinity, alkaline pH, and extreme temperature fluctuations create a unique environment for microbial life. These parameters serve as crucial factors in the survival and adaptation of halophilic archaea, making El-Hamra Lake an ideal location for studying these microorganisms and their potential applications in various fields. A total of ten samples were collected, each in sterile 100 mL containers. Seven samples were taken from the lake's surface, while the remaining three samples were collected from a depth of 50 cm below the surface. To isolate halophilic archaea, a specific culture medium was employed with the following composition per liter: casamino acids (5 g), KH_2_PO_4_ (1 g), MgSO_4_·7H_2_O (0.2 g), NaCl (200 g), trace metals (1 mL), and Na_2_CO_3_ (18 g). The trace metal solution contained the following components per liter: ZnSO_4_·7H_2_O (0.1 g), MnCl_2_·4H_2_O (0.03 g), H_3_BO_3_ (0.3 g), CoCl_2_·6H_2_O (0.2 g), CuCl_2_·2H_2_O (0.01 g), NiCl_2_·6H_2_O (0.02 g), and Na_2_MoO_4_·H_2_O (0.03 g) [11, 12]. Purified colonies were selected, identified, and stored in glycerol at 4 °C. To screen the isolates’ ability to produce biosurfactants, pre-cultures were prepared for each isolate until their optical density (OD) reached approximately 0.9. One mL of the pre-culture was used to inoculate 100 mL of a basal medium adjusted to pH 11 in 250 mL flasks, which were incubated on a shaker at 200 rpm and 37 °C. The specific culture media employed may favor the growth and isolation of certain halophilic archaea species while inhibiting the growth of others, leading to a biased representation. Additionally, the selectivity of the culture media may not accurately reflect the diversity and abundance of halophilic archaea present in the natural environment. Consequently, relying solely on culture-based methods may result in an incomplete understanding of the true microbiota composition. To overcome these limitations, it is essential to complement culture-based approaches with other techniques, such as molecular methods, to obtain a more comprehensive and accurate understanding of the general microbiota. The cell-free supernatant (CFS) was then tested for biosurfactants production using the following methods:

*Emulsification index:* each isolate (BG1, BG2, BG3, BG4, BG5, BG6, BG7, BG8, BG9, BG10, N1, N2, N3, N4) was subjected to emulsification testing using 4 mL of their respective cell-free supernatant (CFS). The CFS was vigorously mixed with an equal volume of various substances, including used vegetable oil, sesame oil, olive oil, flaxseed oil, sunflower oil, or xylene, in individual test tubes. After allowing the emulsions to stand for 24 h, the stability of the emulsions was evaluated. The emulsification index (E24%) was then calculated using the following equation: [13].

$$E24\,\%=\frac{\mathrm{emulsion\,height }\times \mathrm{ cross\, section \,area}}{\mathrm{total \,volume}}\,\times\,100$$

### Surface tension measurement

The culture was subjected to centrifugation at a speed of 15,000 rpm for a duration of 15 min. The resulting supernatant was then utilized to measure the surface tension utilizing a tensiometer (TDI, Lauda, Germany). The surface tension values were expressed in mN/m, with distilled water serving as the reference negative control [14–16].

### Microscopic examination of the most promising biosurfactants producers N3 and BG1

To examine the samples under a light microscope, a cultured broth sample of the most promising isolates was prepared and treated with the Gram staining technique. Archaea exhibit a diverse range of cell wall components, such as pseudopeptidoglycan and S layer proteins, making the Gram staining technique ineffective for their identification. However, in this study we employed alternative methods and staining techniques tailored to the unique cell wall structures of archaea. Techniques like electron microscopy and molecular biology were further refined to accurately identify the targeted archaeal species, compensating for the limitations of Gram staining. These advancements enabled a more comprehensive and precise analysis of the archaeal microbiota under investigation. For scanning electron microscopy (SEM) analysis, a cultured broth was coated with a thin layer of gold using a sputtering device (JFC-1100 E, JEOL, USA) for 12 min. The SEM examination was conducted at 20 kV using a JSM 5300 microscope from JEOL, USA, located at the Faculty of Science, Alexandria University, Egypt [17].

### Molecular identification

For the purpose of molecular identification, a fast DNA extraction technique was utilized in this research. This approach involved the rapid disruption of archaeal cells obtained from individual colonies. Following that, a polymerase chain reaction (PCR) was performed to amplify the 16S rRNA gene from the isolates' genomes using universal primers designed to target approximately 1500 base pairs of this gene. The resulting PCR products were then subjected to sequencing, and the BLAST program was employed to compare them to known sequences and determine their similarity [18].

### Optimization of emulsifiers production using Plackett–Burman design

In this study, the Plackett–Burman design (PBD) was used as a statistical method to evaluate how various culture factors, such as medium components and physical parameters. In the experimental design of the study, several specific factors were considered, and a more detailed description of each factor and its rationale for selection is necessary. The factors include oil, NaCl, casamino acids, pH, and inoculum size. The selection of these factors is significant in relation to biosurfactants production. For example, oil serves as a carbon source for biosurfactant production, NaCl mimics the salinity conditions of the natural environment for halophilic archaea, casamino acids provide essential nutrients, pH affects enzyme activity and microbial growth, and inoculum size influences the rate of biosurfactant production. The specific range of values chosen for each factor was carefully determined to avoid arbitrariness and ensure relevance to the targeted parameter range, thus enhancing the validity and applicability of the proposed experimental conditions. affect the production of biosurfactants by the haloalkaliphilic archaeon (*Natrialba* sp. BG1 & N3). Each factor was tested at two levels: ‘−1’ representing the low level and ‘+1’ representing the high level. This resulted in an experimental matrix consisting of eight trials conducted in 100 mL medium using 250 mL flasks. The emulsification index % (E24%) was measured as the response variable. The PBD is based on a first-order model equation: Y = β0+∑ βi xi, where Y is the response variable, β0 is the intercept, βi represents the regression coefficients, and xi represents the coded levels of the factors. Before the optimization process, a pre-optimization step was carried out. During this step, a medium formula was prepared by setting the most significant factors at their optimal levels determined by the PBD. Factors with a negative effect value were set to their ‘−1’ coded values, while factors with a positive effect value were set to their ‘+1’ coded values. The purpose of this pre-optimization step was to validate the results obtained from the PBD and establish an optimized formula for the subsequent optimization phase [19].

### Response surface

The focus of this optimization step was to identify the most effective variables for achieving the optimal yield of biosurfactants, as measured by the response variable E24%. Each variable was assigned low, middle, and high levels represented by ‘−1’, ‘0’, and ‘ +1’, respectively. The equation used to assess the influence of the three factors on biosurfactants production was as follows:

$${\text{Y }}{\mathbf{ = }}\,\beta_{{0}} { + }\beta_{{1}} \left( {{\text{X}}_{{1}} } \right){ + }\beta_{{2}} \left( {{\text{X}}_{{2}} } \right){ + }\beta_{{3}} \left( {{\text{X}}_{{3}} } \right){ + }\beta_{{{12}}} \left( {{\text{X}}_{{1}} {\text{X}}_{{2}} } \right){ + }\beta_{{{13}}} \left( {{\text{X}}_{{1}} {\text{X}}_{{3}} } \right){ + }\beta_{{{23}}} \left( {{\text{X}}_{{2}} {\text{X}}_{{3}} } \right){ + }\beta_{{{11}}} \left( {{\text{X}}_{{1}} } \right)^{{2}} { + }\beta_{{{22}}} \left( {{\text{X}}_{{2}} } \right)^{{2}} { + }\beta_{{{33}}} \left( {{\text{X}}_{{3}} } \right)^{{2}}$$

In this equation, Y represents the predicted response, which is the percentage of emulsifier production. β0 represents the constant term, and β1, β2, β3 are the linear coefficients. Furthermore, β12, β13, and β23 represent the cross-product coefficients, while β11, β22, and β33 represent the quadratic coefficients. To determine the optimal predicted response and coefficients for the variables, Microsoft Excel 2007 was used for the calculations [20, 21].

### Recovery of the biosurfactants

The culture of BG1 and N3 was subjected to centrifugation (6000 rpm, 30 min) to obtain the biosurfactants produced. The CFS was collected and then acidified to pH 2 using concentrated HCl. It was left to incubate overnight at 4 °C. The resulting precipitate was separated by centrifugation (6000 rpm, 30 min), dried, weighed, and subsequently dissolved in a specific volume of 0.1M Na_2_CO_3_ [22].

### Identification of the extracted biosurfactants

#### Protein, lipid and carbohydrate quantification

The protein, lipid, and carbohydrate contents of the biosurfactants produced by BG1 and N3 were determined using colorimetric methods. To measure the total protein content, 500 µL of biosurfactants, standards, and a blank were mixed with 2.5 mL of alkaline copper solution. The mixture was left to stand for at least 10 min at room temperature. Then, 250 µL of diluted Folin reagent was added and quickly mixed. After 20 min, the samples were read at a wavelength of 750 nm and calculated using a standard curve prepared with different concentrations of human serum ranging from 100 to 500 µg/mL [23]. For measuring the total lipid content, test tubes containing 500 µL of biosurfactants, standards, and a blank were used. To these, 250 µL of concentrated sulfuric acid was added and thoroughly mixed. The test tubes were then heated in boiling water for approximately 5 min. Afterward, 5 mL of phospho-vanillin reagent was added to each tube, mixed well, and incubated at 37 °C in a water bath for 15 min. The tubes were cooled for about 5 min, and within 30 min, the absorbance was measured at a wavelength of 540 nm. Standards were prepared using cholesterol standard with different concentrations ranging from 50 to 200 mg/dL [24]. To determine the carbohydrate content, 600 µL of biosurfactants sample, standards, and a blank were used. To these, 600 µL of 5% w/v phenol was added and mixed well with 3 mL of concentrated sulfuric acid. The test tubes were left at room temperature for 30 min and then measured at a wavelength of 490 nm. Standards were prepared using d-glucose with concentrations ranging from 20 to 100 mg/L [25]. All these measurements were conducted using a double beam meter UV/Vis spectrophotometer (SP-8001) at the marine chemistry lab of the National Institute of Oceanography and Fisheries in Alexandria, Egypt.

#### Fourier transform infrared (FTIR) spectroscopy analysis

FTIR analysis was conducted to partially characterize the molecular structure of the lyophilized biosurfactants. The analysis was performed using a Peak Find-Memory-27 spectrophotometer. Approximately 1mg of the tested material was mixed with 300 mg of pure dry KBr and pressed into discs. Infrared spectra were obtained in the range of 400 to 4000 cm^−1^ using a Bruker Tensor 37 FT-IR spectrophotometer from Bruker, Germany. This analysis took place at the central laboratory of the Faculty of Science, Alexandria University [18].

#### Nuclear magnetic resonance (NMR) spectroscopy-measurement

NMR spectroscopy was employed to investigate the chemical, physical, and biological properties of the lyophilized biosurfactants. The 1H NMR and C NMR spectra were acquired using a Brucker Advance NMR spectrophotometer. The measurements were conducted with a minimum peak distance of 0 Hz and an integral width of 50 Hz in DMSO-d6 as the solvent. The normal 1 setting was used with a reference point at 0.0, start point at 225.81 ppm, and stop point at − 25.80 ppm. This analysis was carried out at the central laboratory of the Faculty of Science, Alexandria University [11].

#### Testing the wound healing activity of the produced biosurfactants

For this study, the W138 cell line (ATCC: CCL-25) was chosen as a specific cell. W138 cells are derived from a human lung carcinoma and have been extensively characterized, making them a reliable model for various research applications. The use of W138 cells ensures consistency and comparability across experiments, enhancing the reliability and relevance of the study findings. WI38 cells (ATCC: CCL-25) were plated in a 24 well plate at a density of 10000 cells per well. The plate was then incubated in a CO_2_ incubator under specific conditions: 37 °C temperature, 5% CO_2_, and 90% relative humidity for 24 h. After the incubation period, the culture medium was replaced with serum-free MEM medium to wash the monolayer of cells. Subsequently, a sterile 200 µl-pipette tip was used to create a scratch (wound) on the cell monolayer. The cell debris resulting from the scratch was washed away using phosphate-buffered saline (PBS) buffer. Next, either 1.5 mL of complete medium alone or complete medium containing 1/10 of the IC50 concentration was added to the plate. The plate was then incubated again for an additional 24 h. The migration of cells into the scratched zone was observed and captured using phase contrast microscopy. The size of the wound was quantified by analyzing the images using Image J software, specifically version 1.49o. [26].

#### Testing the anticancer of the produced biosurfactants

The MTT protocol, as described by Kebbouche-Gana et al. in 2013, was used to determine the cytotoxicity of the samples on cells. A 96-well tissue culture plate was utilized, and each well was seeded with 1 × 105 cells/mL (100 μL/well). The plate was then incubated at 37 ºC for 24 h to allow the cells to form a complete monolayer. Once the cells reached confluence, the growth medium was removed, and the cell monolayer was washed twice with wash buffer (PBS). To prepare the samples for analysis, twofold dilutions were made in RPMI medium containing 2% serum (maintenance medium). Each dilution (0.1 mL) was added to different wells, while three wells were designated as controls, receiving only the maintenance medium. The plate was incubated at 37 ºC for further examination. The cells in each well were visually inspected for any signs of toxicity, such as complete or partial loss of the monolayer, shrinkage, rounding, or cell granulation. A solution of MTT (5 mg/mL in PBS) was prepared, and 20 μL of this solution was added to each well. The plate was placed on a shaking stand at 150 rpm for 5 min to ensure proper mixing of the MTT with the media. Subsequently, the plate was incubated under suitable conditions (37 ºC, 5% CO_2_) for 4 h to allow the cells to metabolize the MTT. After this incubation period, the media was discarded, and if necessary, the plate was gently dried using paper towels to remove any residue. To measure the metabolized formazan (the product of MTT metabolism), it was resuspended in 200 mL of DMSO. The plate was again placed on a shaking stand at 150 rpm for 5 min to ensure effective mixing of the formazan with the solvent. Finally, the optical density of each well was read at 560 nm, and the reading at 620 nm was subtracted to eliminate background noise. The optical density at 560 nm directly correlated with the quantity of cells present in each well [11].

#### Testing the anti-inflammatory activity of the produced biosurfactants

In each well of a 96-well plate, a volume of 50 μL of culture medium containing 3000 WI38 cells was added. To induce inflammation, 50 μL of LPS was added to the plated cells, and the plate was then placed in a CO_2_ incubator. After 24 h, the plate was centrifuged at 1650 rpm for 5 min, and the supernatants were discarded. Subsequently, 100 µL of 1/10 IC50 concentration of the emulsifier extract was added. The control cells consisted of only cell culture medium. The plates were further incubated in the CO_2_ incubator for 72 h. After 72 h of incubation, cell proliferation was assessed using the MTT assay (as described previously). The stimulation index (SI) was utilized to evaluate cell proliferation. The SI was calculated as the mean absorbance of LPS-stimulated cells or LPS-stimulated cells treated with different concentrations of the natural product, divided by the absorbance of control untreated cells. The effective anti-inflammatory concentrations (EAICs) of each extract, which were capable of restoring the abnormal proliferation of LPS-stimulated cells to the normal proliferation of control untreated cells (SI = 1), were determined using the Instate GraphPad software [27].

#### Testing the antioxidant activity

Antioxidant activity of biosurfactants was evaluated at the Biochemistry Department, Faculty of Science, Alexandria University. To conduct the test, 1.9 mL of a prepared phosphomolybdenum reagent was combined and thoroughly mixed with a 100 µL sample of oil or standard (vitamin E) using a vortex. In a similar manner, oil blank tubes were prepared by substituting the phosphomolybdenum reagent with methanol. All the mixture tubes were sealed and placed in a boiling water bath at 95 ° for 90 min. The experiment was performed in triplicate using Wasserman tubes. Following incubation, the absorbance of the resulting greenish-blue complex was measured at 695 nm using a uv/vis spectrophotometer [28]. The total antioxidant capacity of the extracted emulsifier was determined by utilizing the linear regression equation (y = ax−b) obtained from the standard curve of vitamin E. The total antioxidant capacity of the oil was then calculated using the formula:

$${\text{Total antioxidant capacity of oil }}\left( {\text{\% }} \right){ = }\left[ {\left( {{\text{AS }} - {\text{ ASB}}} \right) \, - {\text{b}}} \right]{\text{ / a}}$$

where AS: Mean absorbance of the oil; ASB: Mean absorbance of the oil blank; a: Slope of the vitamin E standard curve; b: intercept of the vitamin E standard curve.

## Results

### Screening for biosurfactants producers

Out of the 14 tested archaeal isolates that exhibited oil displacement, only two isolates, denoted as BG1 and N3, were chosen for further investigation. Table 1 data revealed that BG1 and N3 demonstrated significant potential as biosurfactants producers, as evidenced by their highest E24% (66%) and substantial reduction in surface tension (ST) when tested with various oils. Additionally, these isolates caused blood hemolysis.

**Table 1:** Emulsification index (%) for strains BG (1–10) and N (1–4) using different oils

Strains	Xylene	Used oil	Sesame oil	Olive oil	Flax seed oil	Sunflower oil
**BG1**	**66**	**50**	**66**	**66**	**66**	**33**
BG2	50	16	50	50	16	8
BG3	50	16	50	50	16	0
BG4	33	0	33	50	33	8
BG5	50	16	50	50	50	8
BG6	33	16	33	33	50	16
BG7	33	16	33	33	33	8
BG8	50	16	50	50	16	8
BG9	33	0	33	33	33	0
BG10	33	16	33	33	16	0
N1	16	0	16	0	33	0
N2	0	0	50	0	50	0
**N3**	**66**	**33**	**66**	**66**	**66**	**50**
N4	0	0	50	0	33	0

### Molecular identification, phenotypic characterization and growth pattern of *Natrialba* sp. BG1 and N3

**Fig. 1:**
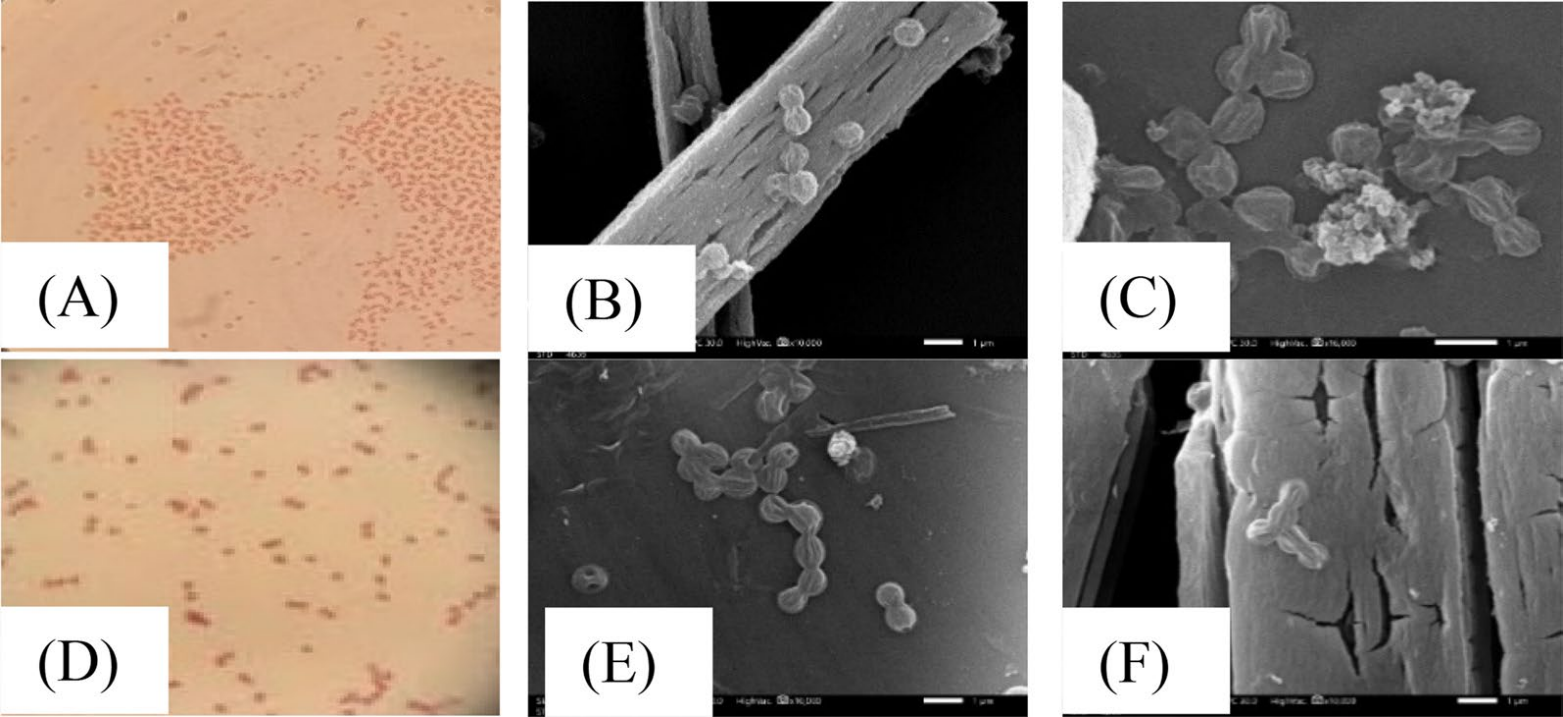
Microscopic examination of the two potential isolates. Above: *Natrialba* sp. BG1 and down: *Natrialba* sp. N3 where **A** and **E** are Gram stain reaction. SEM micrographs of BG1: **B** and **C** with magnification X10,000, and X15,000, respectively. SEM micrographs of N3: **E** and **F** with magnification X10,000 and X15,000

The selected archaeal strains were obtained from El-Hamra lake in Wadi El-Natrun and were identified as *Natrialba* sp. BG1 and *Natrialba* sp. N3. The 16s rRNA gene sequences of both strains were deposited in GenBank. By comparing the previously submitted 16s rRNA sequence results (ac: OR455101, OR689350), a phylogenetic tree was constructed, revealing that *Natrialba* BG1 and N3 were closely related to the cluster of *Natrialba* sp. strains. *Natrialba* sp. BG1 and N3 are appeared spherical under light microscope that produce small, smooth, orange-pigmented colonies on solid medium after being incubated for one week at 37 °C (as depicted in Fig. 1A and D, respectively). Scanning electron microscopy (SEM) images revealed that *Natrialba* sp. BG1 (Fig. 1B, C) and *Natrialba* sp. N3 (Fig. 1E, F) both exhibit a cocci-shaped cell morphology.

### Optimization of biosurfactants production using experimental designs

To conduct the screening phase, five factors were selected at high and low levels in order to optimize biosurfactants production. The design matrix, presented in Table 2, encompasses these levels (1 for high, −1 for low) along with the corresponding response results, namely the emulsification index percentage (E24%), which serves as an indicator of the actual and predicted biosurfactants production. The main effect of each variable on the emulsification index percentage (E24%) was determined by calculating the average difference between the measurements obtained at the high (1) and low (−1) levels.

**Table 2:** PB experimental design for the factors influencing biosurfactants production by *Natrialba* sp. BG1 and N3

Trials	Variables					Response*E24%					
	X1	X2	X3	X4	X5	Actual		Predicted		Residual	
						BG1	N3	BG1	N3	BG1	N3
1	1	− 1	− 1	1	− 1	20	20	20	20	7.1	0
2	1	1	− 1	− 1	1	60	60	55	55	5	5
3	1	1	1	− 1	− 1	40	40	45	45	− 5	− 5
4	− 1	1	1	1	-1	1	1	1	1	5	5
5	1	− 1	1	1	1	40	40	40	40	0	0
6	− 1	1	− 1	1	1	1	1	6	6	− 5	− 5
7	− 1	− 1	1	− 1	1	60	20	60	40	0	0
8	− 1	− 1	− 1	− 1	− 1	40	40	40	20	0	0

Variables	Code	Coded and actual levels	
		− 1	+ 1
Oil (g%)	X1	0.3	0.6
NaCl (g%)	X2	20	30
Casamino acids (g%)	X3	0.3	0.6
pH	X4	10	12
Inoculum size (mL%)	X5	2	4

Triple tests were performed, and the average of three reading was considered as the final

### Statistical analysis of the PBD

The PBD (Plackett–Burman Design) employed in this study is a two-level experimental design that utilizes a linear polynomial correlation model to establish the relationship between the five factors and the corresponding response. The correlation models for *Natrialba* sp. BG1 and N3 are as follows:

$${\text{BG1 }}:{\text{ Y }} = { 32}.{75 } + { 7}.{\text{25X1 }} - { 7}.{\text{25X2 }} + { 2}.{\text{5X3 }} - { 17}.{\text{25X4 }} + { 7}.{\text{5X5}}$$

$${\text{N3}}:{\text{ Y }} = {\text{ 27}}.{\text{25 }} + {\text{ 12}}.{\text{25X1 }} - {\text{ 2}}.{\text{25X2 }} + {\text{ 2}}.{\text{5X3 }} - {\text{ 12}}.{\text{25X4 }} + {\text{ 7}}.{\text{5X5}}$$

**Fig. 2:**
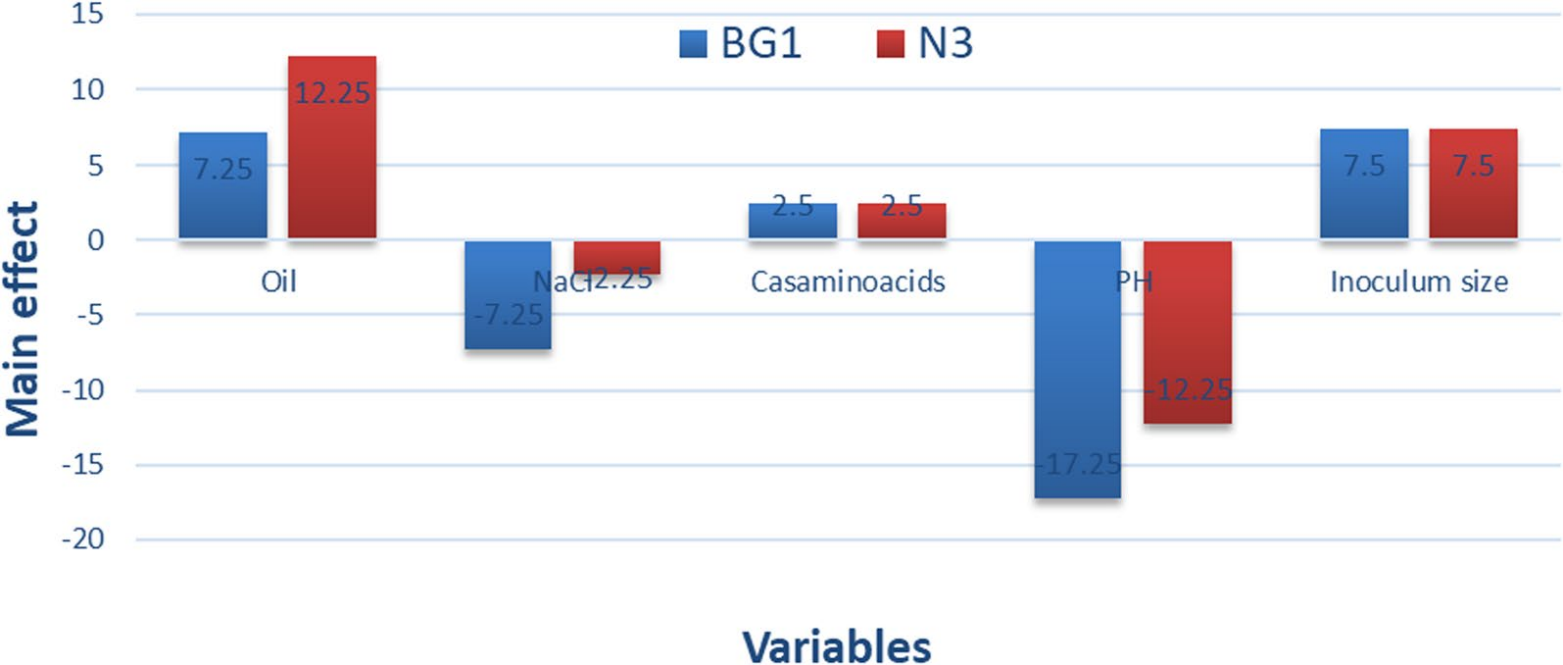
Main effect of the factors influencing the biosurfactants production by *Natrialba* sp. BG1 and N3 based on PBD

The data underwent variance analysis using Microsoft Excel tools and the JMP program. The R2 value, a measure of model quality, was employed in this study. The obtained R2 values for *Natrialba* sp. BG1 and N3 were 0.973 and 0.967, respectively, indicating a high degree of fitness for the predicted models. Based on the main effect results illustrated in Fig. 2, the significant factors influencing biosurfactants production, in descending order, for BG1 were inoculum size, oil, casamino acids, NaCl, and pH. For N3, the significant factors were oil, inoculum size, casamino acids, NaCl, and pH. Through regression coefficient analysis of these five factors, it was observed that oil, casamino acids, and inoculum size exhibited a positive effect on biosurfactants activity. Conversely, NaCl and pH had a negative effect on biosurfactants production for both archaeal strains. Consequently, NaCl, pH, and inoculum size were identified as the most significant factors for further optimization steps.

### Optimization of the culture conditions using BBD

In order to determine the optimal region for biosurfactants production, the three most significant independent variables (oil, pH, and inoculum size) were explored at three levels. Table 3 presents the design pattern for these variables, along with the response results (residual, predicted, and actual) for each trial in the design pattern. The response measured was the Emulsification index percentage (E24%). The coded values (1, 0, −1) of the selected factors are also displayed in Table 3. To predict the optimal point, a second-order polynomial function was fitted to the experimental response results using a non-linear optimization algorithm. The fitted equations for BG1 and N3 are as follows:

$${\text{BG1:Y = 50}} - {12}{\text{.25X1}} - {1}{\text{.625X2}} - {4}{\text{.625X3 + 9}}{\text{.25X1X2 + 9}}{\text{.25X1X3}} - {\text{6X2X3}} - {12}{\text{.25X1}}^{{2}} - {15}{\text{.5X2}}^{{2}} - {15}{\text{.5X3}}^{{2}}$$

$${\text{N3:Y = 45}}{.6666} - {11}{\text{.625X1}}\, - {0}{\text{.625X2}} - {4}{\text{.5X3 + 6X1X2 + 9}}{\text{.25X1X3}} - {10}{\text{.25X2X3}} - {13}{\text{.5833X1}}^{{2}} - {12}{\text{.0833X2}}^{{2}} - {12}{\text{.8333X3}}^{{2}}$$

**Table 3:** BB factorial experimental design for the biosurfactants production by *Natrialba* sp. BG1 and N3

Trials	Variables			Response*E24%					
	X1	X2	X3	Actual		Predicted		Residual	
				BG1	N3	BG1	N3	BG1	N3
1	0	0	0	50	46	50	45.67	0	0.33
2	0	0	0	50	45	50	45.67	0	− 0.67
3	0	− 1	1	25	30	22	27.13	3	2.88
4	0	1	-1	25	32	28	34.88	− 3	− 2.88
5	− 1	− 1	0	50	43	45.38	38.75	4.63	4.25
6	0	0	0	50	46	50	45.67	0	0.33
7	1	0	− 1	13	10	5.38	2.88	7.63	7.13
8	0	1	1	13	11	6.75	5.38	6.25	5.63
9	0	− 1	− 1	13	10	19.25	15.63	− 6.25	− 5.63
10	− 1	0	1	13	10	20.63	17.13	− 7.62	− 7.13
11	− 1	0	− 1	50	46	48.38	44.63	1.63	1.38
12	1	0	1	13	11	14.63	12.38	− 1.62	− 1.38
13	1	1	0	13	10	17.63	14.25	− 4.62	− 4.25
14	1	− 1	0	1	1	2.38	2.5	− 1.37	− 1.5
15	− 1	1	0	25	26	23.63	24.5	1.38	1.5

Variables	Code	Coded level and actual level		
		− 1	0	+ 1
Inoculum size (mL%)	X1	4	5	6
pH	X2	10	11	12
NaCl (g%)	X3	20	25	30

Triple tests were performed, and the average of three reading was considered

**Fig. 3:**
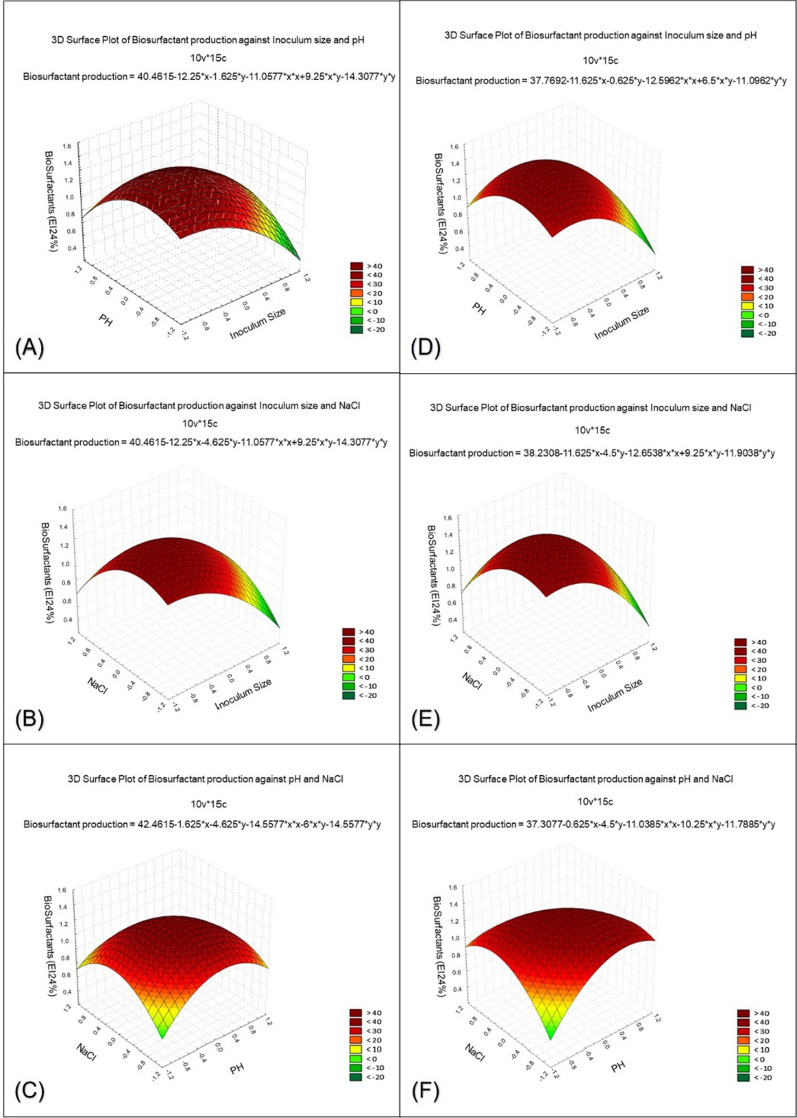
Three-dimensional surface showing the relationships between the tested factors and the biosurfactants as response in the form of Emulsification index E24% produced by *Natrialba* sp. BG1 and N3 **A** showed that at high inoculum size and middle pH gave the highest biosurfactants production level. **B** showed that at high inoculum size value and middle value of NaCl gave the highest biosurfactants production level. **C** showed foci for maximum level of biosurfactants production at middle both NaCl and pH for Natrialba sp. BG1 also **D** indicated that at high inoculum size value and middle pH values the maximum production was achieved. **E** showed the maximum response at high inoculum size and middle value of NaCl (**F**) showed foci for maximum level of biosurfactants production at middle values of both NaCl and pH

The multiple correlation coefficients R and the determination coefficient R^2^ were used as correlation measures for estimating the regression equation on the model level. The values of R^2^ were 0.941 and 0.943 for BG1 and N3, respectively, indicating a strong correlation between the actual and predicted values. Figure 3 illustrates the simultaneous effects of the three most significant independent variables on each response using three-dimensional graphs generated by STATISTICA 7.0 software. Additionally, a profile was created to determine the optimal levels of the three selected variables. The optimal levels, obtained from the maximum point of the polynomial model using the SOLVER function in Microsoft Excel tools and JMP-program, were found to be: inoculum size 2% (− 0.5), pH 11 (0), and NaCl 250 g/L (0) for *Natrialba* sp. BG1, and inoculum size 2.2% (− 0.55813), pH 10 (0), and NaCl 100 g/L (− 0.5) for *Natrialba* sp. N3. The predicted response values for BG1 and N3 were 53.0625 and 49.74632, respectively. Figure 3 demonstrates the amount of biosurfactants obtained after extraction using the basal medium (0.357, 0.276) g/L and the optimized medium (0.625, 0.557) g/L for BG1 and N3, respectively.

Figure 3 displays the three-dimensional surface plots illustrating the relationships between the tested factors and the biosurfactants production, indicated by the Emulsification index (E24%), for *Natrialba* sp. BG1 and N3. The plots reveal that the highest levels of biosurfactants production were observed when using a high inoculum size and a moderate pH (A and D). Similarly, for both strains, the highest biosurfactants production was achieved with a high inoculum size and a moderate NaCl concentration (B and E). Furthermore, (C and F) exhibit focal points indicating the maximum levels of biosurfactants production at moderate.

### Characterization of the produced biosurfactants

#### Chemical analysis of the biosurfactants produced by *Natrialba* sp. BG1 and N3

**Fig. 4:**
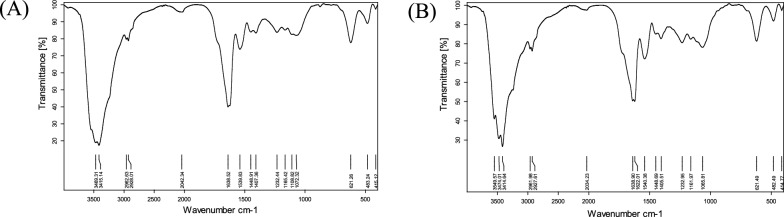
FT-IR Spectra for *Natrialba* sp. BG1 biosurfactants (**A**) and *Natrialba* sp. N3 biosurfactants (**B**)

**Fig. 5:**
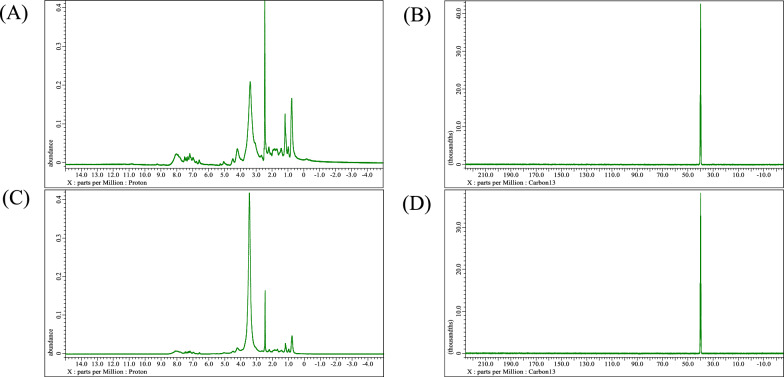
**A** C13 NMR for *Natrialba* sp. BG1 biosurfactants **B** 1H NMR for *Natrialba* sp. BG1 biosurfactants, **C** C13 NMR for *Natrialba* sp. N3 biosurfactants and **D** 1H NMR for *Natrialba* sp. N3 biosurfactants

The biosurfactants produced by *Natrialba* sp. BG1 was found to contain 56.8% protein, 1.8% carbohydrate, and 5.96% lipid. On the other hand, the biosurfactants produced by *Natrialba* sp. N3 had protein, carbohydrate, and lipid contents of 68, 4.82, and 9.44%, respectively. This comparative analysis highlights the strain-specific variations in biosurfactant composition within *Natrialba* sp. BG1 and N3. The differences in protein, carbohydrate, and lipid content suggest that these strains may produce biosurfactants with distinct molecular profiles and potentially different functional properties. The FTIR analysis presented in Figure 4 unveiled several characteristic bands and peaks. The FTIR analysis revealed characteristic bands and peaks, indicating the presence of specific functional groups in the biosurfactants. For example, the broad band at 3469.31 cm^−1^ suggested strong hydrogen bonds associated with NH stretching mode, while the peaks at 2962.63 cm^−1^ corresponded to aliphatic chains (–CH3, –CH2). The absorbance signals at 2360.88 and 2075.86 cm^−1^ indicated the presence of R2C=N=N stretch, and the peak at 1638.52 cm^−1^ suggested a linkage between amides, indicating the significant presence of the peptide group. The moderate intensity peak at 1165.42 cm^−1^ indicated vibrations of carboxylic acids, aldehydes, and ketones. The NMR analysis further confirmed the composition of the biosurfactants, with specific peaks corresponding to methyl hydrogens (R–CH3), alkyl hydrogens (R–CH2–R), and hydrogens associated with a C–O bond. The C13 NMR analysis displayed peaks corresponding to carbon atoms of R–CH3, carbon atoms of R–CH2–R, and carbon atoms attached to oxygen. Overall, the integrated analysis of FTIR and NMR spectra confirms that the extracted biosurfactant is a glycolipid consisting of a hydrophilic disaccharide moiety and a hydrophobic octadecanoicaci The NMR of the biosurfactants is depicted in Fig. 5.

#### Wound healing ability of the biosurfactants produced by *Natrialba* sp. BG1 and N3

**Fig. 6:**
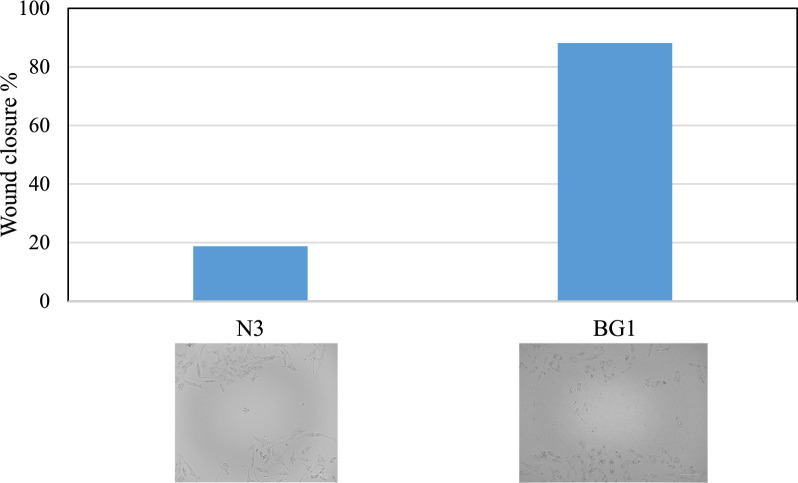
Wound closure % of *Natrialba* sp. N3 and BG1 biosurfactants

In a histopathological examination, a wound was created and left to heal naturally for 24 h. When comparing the use of biosurfactants to the control group, it was observed that complete re-epithelization occurred in case of biosurfactants more faster than the control. Additionally, there was evident growth of fibrous connective tissues in the dermis layer. However, when comparing the effects of *Natrialba* sp. BG1 biosurfactants and *Natrialba* sp. N3 biosurfactants, it was found that *Natrialba* sp. BG1 resulted in a higher wound closure rate compared to *Natrialba* sp. N3. Specifically, *Natrialba* sp. BG1 accelerated wound closure by 87% faster, whereas *Natrialba* sp. N3 biosurfactants only accelerated wound closure by 18% compared to the control group. These findings demonstrate that the wound healing effect of *Natrialba* sp. BG1 biosurfactants was superior to that of *Natrialba* sp. N3 biosurfactants, as depicted in Fig. 6.

#### Investigation of the biosurfactants activity against four human cancer cell lines

**Fig. 7:**
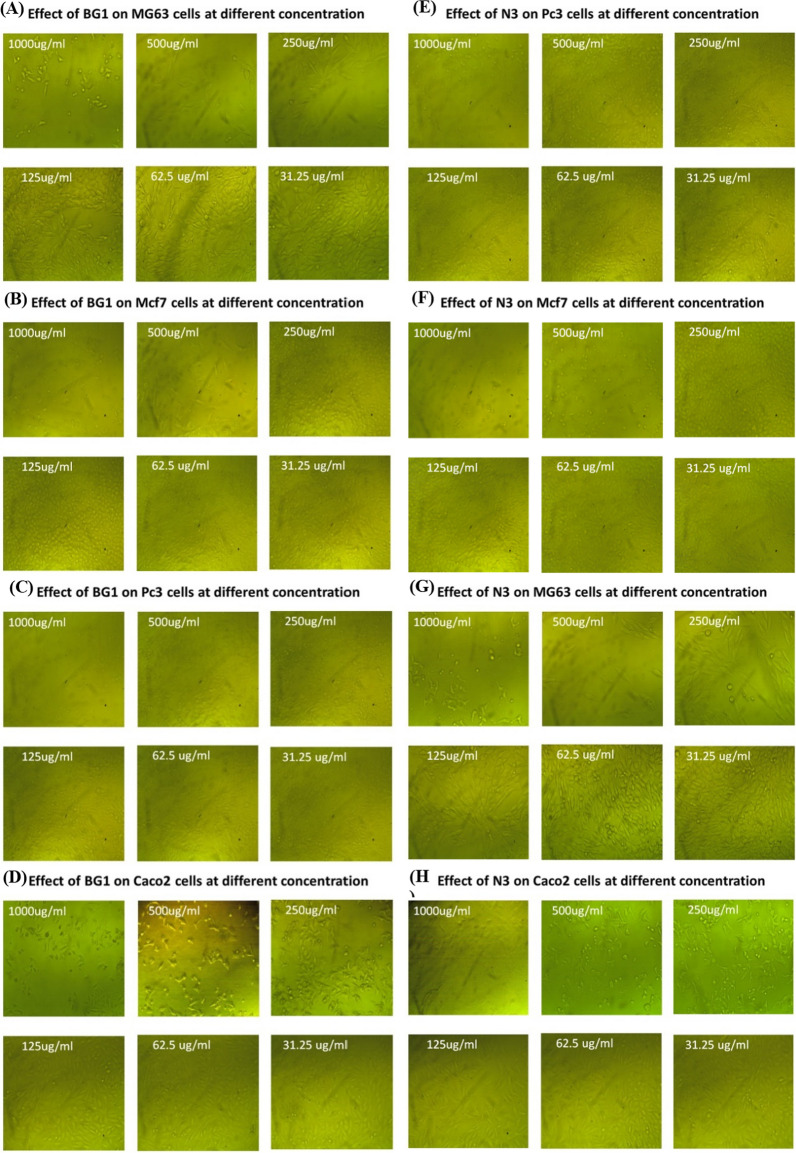
Effect of biosurfactants produced by *Natrialba* sp. BG1 (**A**, **B**, **C** & **D**) on MG63, MCF7, PC3 and MG63 cells at different concentration respectively, and (**E**, **F**, **G** & **H**) showed effect of *Natrialba* sp. BG1 biosurfactants on MG63, MCF7, PC3 and MG63 cells at different concentration respectively

**Fig. 8:**
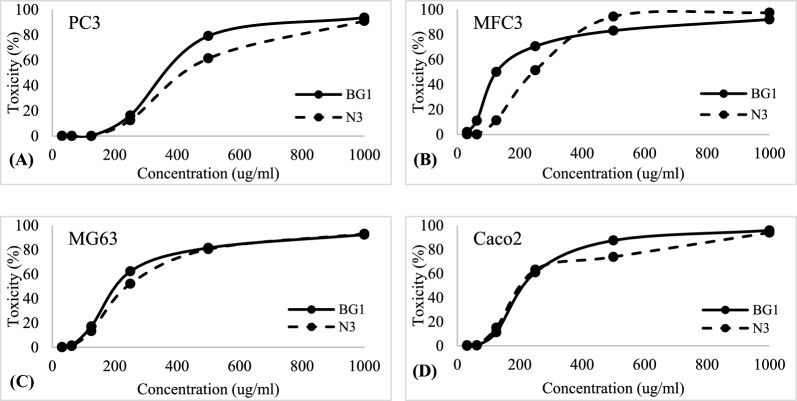
Toxicity of the biosurfactants produced by *Natrialba* sp. BG1 and N3 on different cell lines (**A**) PC3, (**B**) MFC3, (**C**) MG63 and (**D**) Caco2

Before evaluating the anticancer properties of the produced biosurfactants, its impact on normal cell lines was examined. The cytotoxicity of *Natrialba* sp. BG1 and N3 biosurfactants was tested on normal cell lines, alongside Doxorubicin as a positive control, using varying concentrations. Furthermore, different cancer cell lines including PC3 (Prostate human epithelial cells), MFC3 (Mammary gland human epithelial cells), MG63 (Bone human fibroblast cells), and Caco2 (Colon human epithelial cells) were exposed to the biosurfactants, and their effects were assessed through the MTT assay. The findings indicated that the tested biosurfactants exhibited cytotoxic effects on the cancer cell lines at different concentrations. Due to comparative analysis of the biosurfactants produced by *Natrialba* sp. BG1 and N3, along with their anticancer activity, provides valuable insights into their potential as anticancer agents. The differences in the chemical composition of the biosurfactants, as mentioned earlier, may contribute to variations in their efficacy or potency. Notably, the high IC50 values observed in normal cells indicated the biosurfactants' safety profile, while the low IC50 values in cancer cell lines indicated their potent anticancer activity. Specifically, *Natrialba* sp. BG1 and N3 biosurfactants displayed a toxicity effect against PC3, resulting in 93.69 and 91.02% inhibition, respectively, at a concentration of 1000 µg/mL (Figs. 7A and E, 8A) and Table 4. Similarly, they exhibited inhibition against MFC3 at the same concentration, with *Natrialba* sp. BG1 and N3 biosurfactants showing 92.1 and 97.59% inhibition, respectively (Figs. 7B and F, 8B) and Table 5. Regarding MG63 and Caco2, *Natrialba* sp. BG1 and N3 extracts demonstrated inhibitory effects of 92.45 and 93.1, and 95.86 and 94%, respectively, at the same concentration (Fig. 7C, D, G and H) and Tables 6 and 7.

**Table 4:** Cytotoxicity effect on PC3

Toxicity of BG1 and N3 on PC3		
Concentration (µg/mL)	BG1	N3
1000	93.692	91.020
500	79.150	61.498
250	16.338	12.527
125	0.175	0.0438
62.5	0.131	0.131
31.25	0.175	0.2628

**Table 5:** Cytotoxicity effect on MFC3

Toxicity of BG1 and N3 on MFC3		
Concentration (µg/mL)	BG1	N3
1000	92.107	97.596
500	83.173	94.471
250	70.633	51.482
125	50.120	11.378
62.5	11.098	0.080
31.25	2.043	0.1602

**Table 6:** cytotoxicity effect on MG63

Toxicity of BG1 and N3 on MG63		
Concentration (µg/mL)	BG1	N3
1000	92.457	93.118
500	81.613	80.535
250	62.357	52.207
125	17.171	13.278
62.5	1.564	1.078
31.25	0.3128	0

**Table 7:** Cytotoxicity effect on Caco2

Toxicity of BG1 and N3 on Caco2		
Concentration (µg/mL)	BG1	N3
1000	95.866	94.019
500	87.555	73.879
250	61.038	63.281
125	11.214	15.084
62.5	0.4398	0.3078
31.25	0.484	0

#### Anti-inflammatory activity of *Natrialba* sp. N3 and BG1 biosurfactants

**Fig. 9:**
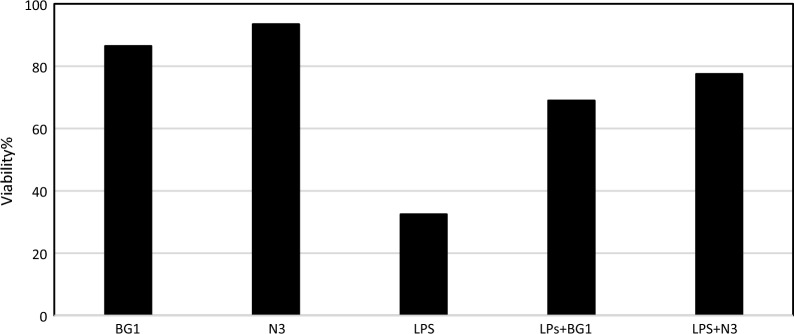
Anti-inflammatory effect of *Natrialba* sp. N3 and BG1 biosurfactants using LPS

To assess the cytotoxicity of *Natrialba* sp. BG1 and N3 biosurfactants on WI38 cells, the viability of the cells was evaluated using the 3-(4,5-dimethylthiazol-2-yl)-2,5-diphenyl-tetrazolium bromide (MTT) assay. WI38 cells were exposed to the tested *Natrialba* sp. BG1 and N3 biosurfactants, along with LPS, and incubated for 72 h to measure absorbance. The results revealed no significant variations between the tested biosurfactants and the untreated control in WI38 cells, as depicted in Fig. 9. Thus, the biosurfactants demonstrated no cytotoxicity at the same concentration. Furthermore, to investigate the impact of *Natrialba* sp. BG1 and N3 biosurfactants on W138 cell proliferation, the findings indicated that both extracts induced cell division and enhanced viability in the presence of LPS, demonstrating an anti-inflammatory effect. Notably, *Natrialba* sp. N3 biosurfactants exhibited a slightly stronger anti-inflammatory effect compared to *Natrialba* sp. BG1 biosurfactants.

#### Antioxidant activity of both *Natrialba* sp. N3 and BG1 biosurfactants

**Fig. 10:**
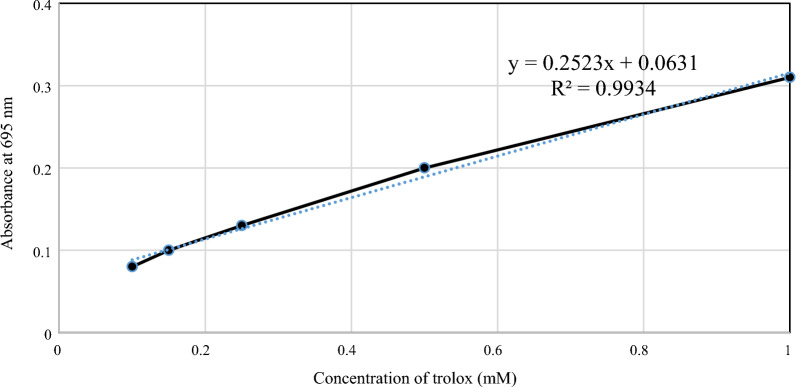
Second order equation to calculate the equivalent antioxidant amount percentage in both *Natrialba* sp. BG1 and N3 biosurfactants

Table 8 and Fig. 10 present the antioxidant capacity of the biosurfactants solutions. The results of this study indicate that both *Natrialba* sp. N3 and BG1 biosurfactants demonstrated antioxidant capacity at a concentration of 75 µg/mL. Specifically, the antioxidant capacity of *Natrialba* sp. N3 biosurfactants was determined to be 8.820187607 mM trolox equivalent/g extract at the same concentration, while the BG1 biosurfactants exhibited an antioxidant capacity of 5.173734972 mM trolox equivalent/g extract when assessed using the phosphomolybdenum reagent.

**Table 8:** Antioxidant capacity of biosurfactants produced by *Natrialba* sp. BG1 and N3

Name of samples	Conc of samples (mg/mL)	Absorbance at ???	Conc of antioxidant in sample (mg/mL)	Conc of antioxidant in sample (mM trolox equivalent/g extract)	Conc of antioxidant in sample (%)
N3	75	0.23	0.662	8.820	11.760
BG1	75	0.161	0.388	5.174	6.898

## Discussion

This study focused on the production of biosurfactants by extremely halophilic archaea, which have the ability to adapt to high temperatures and NaCl concentrations, enhancing their stability in organic solvents. Fourteen haloarchaeal strains isolated from El-Hamra Lake, Wadi El-Natrun, Egypt, were screened for biosurfactant production under extreme hypersaline conditions. Two halophilic archaea isolates, *Natrialba* sp. BG1 and N3, were identified and characterized for their biosurfactant production. Statistical optimization experiments identified the significant factors affecting biosurfactant production, such as inoculum size, oil, casamino acids, NaCl, and pH. The results revealed that oil, casamino acids, and inoculum size had a positive effect on biosurfactant activity, while NaCl and pH had a negative effect. The study integrated existing literature on biosurfactants, highlighting the importance of carbon sources, such as glucose and glycerol, in biosurfactant production, as well as the potential of biosurfactants for facilitating the uptake and biodegradation of aromatic hydrocarbons at high NaCl concentrations. The findings contribute to our current understanding of biosurfactant properties and their potential applications in various industries. Different selected variables were studied to assume their effect on the biosurfactants production using PBD. The such statistical design was previously applied to study the biosurfactants production by *Natrialba* sp. M6 which is isolated from Wadi El-Natrun, Egypt. [18]. Which found ammonium nitrate and glucose were more effective when used in negative levels than glycerol and pH when used at positive. Thus, both pH and glycerol were chosen for the next optimization step. Heryani and Putra, described that a high concentration of glucose caused an inhibition for the biosurfactants production due to the acidic metabolites’ formation [29]. Also many researchers interested to study the production of biosurfactants by halophilic archaeon *Natrialba* sp. C215 and *Bacillus* sp. which reported the ability of these organisms to produce biosurfactants and its using to facilitate the aromatic hydrocarbons uptake and their biodegradation at high NaCl concentrations, also the effect of various carbon sources in the production medium (glycerol and glucose) were investigated because the carbon source type is very important in the biosurfactants production. The partially purified produced biosurfactants were extracted and identified using 1HandC13 NMR analysis as reported by Jerković [30], and the results indicated that the recovered extract contained different groups 1H NMR showed peaks at chemical shifts 0.8 which confirmed the presence of methyl hydrogens (R–CH3). A peak obtained at 1.1 shows the presence of alkyl hydrogens (R–CH2–R). Peaks obtained in the range 3.1–3.7 confirmed the presence of hydrogens associated with a C-O bond. Peaks within the range of 3.0 to 3.7 also correspond to hydrogens of alcohol and ester bonds. But C13 NMR of the extracted biosurfactants showed peaks at chemical shift 13.9 which corresponds to C of R-CH3. Peaks between 18 to 30 designate C of R–CH2–R. Carbon attached to oxygen is designated by peaks between shifts 50 to 78 ppm. In addition, total proteins, carbohydrates, and lipids measurement of both BG1 and N3 analyses indicated that the percentages of protein, carbohydrate, and lipid contents of BG1, biosurfactants were found to be 56.8, 1.8, and 5.96%, respectively. The percentages of protein, carbohydrate, and lipid contents of N3, biosurfactants were found to be 68, 4.82, and 9.44%, respectively, which mean that the produced biosurfactants of the two strains is lipoprotein. Also, the FTIR indicated the presence of characteristic broadband at 3469.31 cm^−1^ for NH stretching mode, indicating a strong hydrogen bond. The week peak observed at 2962.63 cm^−1^ was a characteristic band of aliphatic chains (–CH3, –CH2) stretching vibrations. Absorbance signals detected at 2360.88 and 2075.86 cm^−1^ may be due to the presence of R2C=N=N stretch. A strong band peak observed at 1638.52 cm^−1^ was indicated by a definite linkage between the amides and considered a significant presence of the peptide group in the molecule. A moderate intensity peak in the region of 1165.42 cm^−1^ was assigned by CO-extending vibrations of carboxylic acids, aldehydes, and ketones. Extended vibrations observed at 621.26 cm^−1^ may be alkene. The composition of biosurfactants produced by different microbial species has been a subject of interest in previous studies. Consistent with the findings of Habib et al. [31], our study revealed a significant proportion of protein (25%) and lipid (64%) in the biosurfactants produced by *Rhodococcus* sp. ADL36, indicating their potential as lipopeptides. This aligns with the work of Hegazy et al. [14], who reported that biosurfactant components are predominantly composed of lipids (41%) and proteins (31%). These consistent findings across different microbial species underscore the recurring presence of specific biomolecules in biosurfactants and contribute to our understanding of their composition and potential applications. In terms of wound healing activity, our study showed that the biosurfactants extracted from *Natrialba* sp. BG1 exhibited a remarkable wound closure acceleration of 87% within a 24 h period. In contrast, the biosurfactants from N3 demonstrated a lower wound closure rate of only 18% within the same time frame. These results are in agreement with the findings of Ohadi et al. [26], where biosurfactants produced by *Acinetobacter junii* B6 were found to enhance the re-epithelialization process, reduce neutrophilic inflammation, promote hair follicle detection, decrease edema, and facilitate the removal of necrotic tissue, ultimately aiding in the maturation of the wound bed. To fully appreciate the significance of these findings, it is crucial to align them with the broader objectives of our research. Exploring the diverse applications of biosurfactants beyond their traditional properties is a key aspect of our study. The consistent presence of lipids and proteins in biosurfactants, as supported by previous research, suggests their potential as lipopeptides with versatile properties. Furthermore, the accelerated wound closure observed with the biosurfactants from *Natrialba* sp. BG1 highlights their potential as wound healing agents. In light of these findings, it is recommended that future research should focus on elucidating the specific mechanisms underlying the wound healing properties of biosurfactants and further exploring their potential applications in various medical and industrial settings. This can include investigating the interactions of biosurfactants with cellular components involved in wound healing processes and conducting in vivo studies to assess their efficacy and safety. By expanding our knowledge in these areas, we can unlock the full potential of biosurfactants and contribute to the development of innovative therapies and sustainable solutions. The BG1 and N3 biosurfactants exhibited anticancer activity against different types of cancer cell lines such as PC3, MFC3, MG63, and Caco2 and the results showed that the produced biosurfactants revealed cytotoxicity against the tested cell lines, the toxicity effect of BG1 and N3 biosurfactants against PC3 was 93.69% and 91.02 cells growth inhibition respectively at a concentration of 1000 µg/mL, while against MFC3 BG1 and N3 biosurfactants showed inhibition at a concentration 1000 µg/mL 92.1 and 97.59% respectively. Also, the inhibition of BG1 and N3 extracts against MG63 was 92.45 and 93.1% and against Caco2 was 95.86 and 94.01% respectively at the same concentration. Furthermore, it is important to discuss the anticancer results in relation to the biosurfactants' chemical composition. The high IC50 values observed in normal cells indicate the biosurfactants' safety profile, suggesting that they may have a selective cytotoxic effect on cancer cells. The low IC50 values in cancer cell lines indicate their potent anticancer activity. Specifically, the *Natrialba* sp. BG1 and N3 biosurfactants displayed significant inhibition against PC3, MFC3, MG63, and Caco2 cancer cell lines at a concentration of 1000 µg/mL. This integrated analysis of the biosurfactants' chemical composition and their anticancer activity can provide a more comprehensive understanding of their potential as anticancer agents. The evidence presented in this study demonstrates the potential of biosurfactants to accelerate the apoptosis process in cancer cell lines [32]. Our research specifically focused on investigating the anticancer activity of biosurfactants produced by various strains of *Bacillus* sp. against breast cancer. The findings revealed significant growth inhibition, apoptosis induction, and suppression of colony formation. These effects can be attributed to the biosurfactants' ability to regulate the cell cycle's regulatory protein, leading to the disruption of cancer cell regulation. Another relevant study conducted by El-Naggar et al. [33] investigated the impact of polysaccharides produced by *Haloarchaea biusiranensis.* The results indicated that these polysaccharides had no effect on normal cells but reduced the viability of cancer cells, including breast cancer cell line MDA-MB468, prostate cancer cell lines DU145, and lung cancer cell line A549. It is important to note that the diversity in methodological approaches across different studies may limit direct data comparability. The emulsifiers cytotoxicity of both tested archaeal strains against W138 cells, and the viability of the cells was studied, and the results revealed the ability of the produced biosurfactants to induce the cell division and viability of the cells, N3 extract showed higher anti-inflammatory activity than of BG1 extract [34]. study indicated the anti-inflammatory activity of the biosurfactants produced by several microorganisms (yeast, *Bacillus subtilis*, *Pseudomonas aeruginosa*, and *Lactobacillus* sp.) and also their anti-viral activity against (COVID-19).The antioxidant potential of the produced biosurfactants of the two BG1 and N3 strains were tested in the assays of the radical scavenging, based on phosphomolybdenum reagent assay. The two extracts exhibited antioxidant activity and were able to scavenge the free radicals and reduce of Mo(VI) to Mo(V) and the subsequent formation of a green phosphate/Mo(V) complex at acidic pH. Villegas et al. [35]. studied the antioxidant activity of the extract of two haloarchaeal strains isolated from Odiel salterns (Southwest Spain) and the results showed that none of produced extracellular extracts have antioxidant capacity, while it was found that the cellular extracts have antioxidant potential and able to scavenge ABTS and DPPH radicals and also can reduce ferrocyanide and chelate copper but cannot chelate iron radicals. The study demonstrates biosurfactant production from halophilic archaea with potential biological activities. However, limitations include a narrow focus on specific strains from a single location, lacking comprehensive characterization of biosurfactants, the need for in vivo studies to evaluate practical applications and efficacy, and the importance of investigating environmental impact and biodegradability. Further research involving diverse strains, detailed characterization, clinical trials, and environmental assessments would address these limitations and enhance the practical applications of halophilic archaea biosurfactants.

## Conclusion 

This study presents a novel investigation on biosurfactant production from extremely halophilic archaea isolated from El-Hamra Lake, Wadi El-Natrun, Egypt, specifically *Natrialba* sp., BG1, and N3 strains. The study identifies optimal conditions for maximum biosurfactant production and reports promising yields using both basal and optimized mediums. The unique properties and potential applications of these biosurfactants are highlighted. However, to facilitate practical applications, further research is needed to address existing gaps. Specifically, future studies should focus on exploring diverse applications beyond traditional uses, and linking the recommended optimization parameters to the significance of these biosurfactants. Additionally, efforts should be directed towards understanding the detailed mechanisms of action, conducting in vivo studies, and assessing the environmental impact and biodegradability of these biosurfactants. By addressing these gaps, the practical applications of the biosurfactants obtained in this research can be better realized.

## Data Availability

All the data generated during this study are included in this published article.
